# A comparative study of the effect of drilling depth on generation of compressive force by headless compression screws using conical and cylindrical type of drill bit

**DOI:** 10.1186/s13018-018-1044-2

**Published:** 2019-01-04

**Authors:** Hyung-Sik Kim, Ho-Jung Kang, Yun-Rak Choi, Won-Taek Oh, Il-Hyun Koh

**Affiliations:** Department of Orthopaedic Surgery, Yonsei University College of Medicine, 50 Yonseiro, Seodaemun-gu, Seoul, Republic of Korea

**Keywords:** Biomechanics, Compression force, Drill bit type, Drilling depth, Headless compression screw

## Abstract

**Background:**

This study was conducted to measure the effect of different drilling depths on compression forces generated by two commonly used headless compression screws using the two different types of drill bit, the Acutrak® mini (conical type drill bit) and the Synthes 3.0 HCS® (cylindrical type drill bit).

**Methods:**

A load cell was placed between two Sawbone blocks, which were 12 mm and 40 mm in thickness, respectively. After placing the guide pin into the center of the block, the drilling depth of the Acutrak® mini and Synthes HCS® screws ranged from 16 to 28 mm and 22 to 28 mm, respectively. The 24-mm screws were inserted and the compression force was measured immediately and at 30 min post-insertion.

**Results:**

The Acutrak® mini generated greater compression force compared to the Synthes 3.0 HCS® when drilled to a depth of less than 24 mm. The compression force of the Acutrak® mini showed a strong inverse correlation with the drilling depth. There was no significant inverse correlation observed between the compression force of the Synthes HCS® and the drilling depth.

**Conclusions:**

If the screw length and the drill depth are the same, the Synthes 3.0 HCS® (cylindrical type drill bit) is safer and easier to use as it has no change in the compression force even when over-drilling because the compression force of the two screws is similar. As for the Acutrak® mini (conical type drill bit), while it is technically demanding due to varying compression force according to the drill depth, it can be used in certain cases because it can give stronger compression force through under-drilling.

## Introduction

Headless compression screws, designed to maintain compression force when the head is inserted into the bone, are commonly used because of minimal risk of tissue irritation and damage to the surrounding articular cartilage [1, 2]. Headless compression screws generate compression force through different pitches and diameters between the leading and the trailing threads [3]. Generally, the leading thread has a wider thread pitch with a smaller diameter, while the trailing thread has a narrower pitch and a larger diameter [4]. The type of the drill bit may vary depending on the diameter difference between the leading thread and the tailing thread, as well as the design of the screw itself. Threadless central shaft screws such as the Synthes 3.0 HCS® (Synthes Inc.®, Westchester, PA, USA), which have threads at both ends and a sparing central shaft, use a cylindrical type drill bit with a constant diameter; these screws are designed to have a self-tapping trailing end. On the other hand, full-threaded variable pitch screws such as the Acutrak® Mini screw (Acumed®, Hillsbro, OR, USA) have threads over the whole length with gradually increasing diameter; these screws require a conical type drill bit. Although threadless central shaft screws can generate consistent compression force along the central shaft, the threadless area may also cause instability because of the relatively small contact area with the surrounding tissue. In contrast, full-threaded variable pitch screws may generate different compression forces along the screw, but can achieve high stability by creating a large total contact area [5, 6].

Although many studies have compared the biomechanical properties of the two types of headless compression screw, the relationship between the compression force and the drilling depth has not been studied, especially when using the two different types of drill bit [4, 7–10]. Variation in compression forces generated by headless compression screws may be due to the type of drill bit, and whether the drill bit is over-drilled or under-drilled. In the clinical setting, if the operating surgeon can estimate the compression force generated by different drilling depths, it may be possible to optimize the interfragmentary compression force by selecting the appropriate drill bit and the proper drill depth.

Therefore, we measured the effect of drilling depth on the compression force generated by two different types of the headless compression screw frequently used for fixation of scaphoid fractures and non-unions: the Synthes 3.0 HCS® and Acutrak® Mini screws.

## Material and methods

### Screws

The Synthes 3.0 HCS® is a titanium headless screw consisting of a threadless central shaft and threads of different pitches at each end. The leading thread has a diameter of 3 mm and a thread pitch of 1.2 mm, and the trailing thread has a larger diameter of 3.5 mm and narrower pitch of 0.6 mm. The drill bit used for the Synthes 3.0 HCS® is a cylindrical type drill bit with a constant diameter of 2.0 mm.

**Fig. 1:**
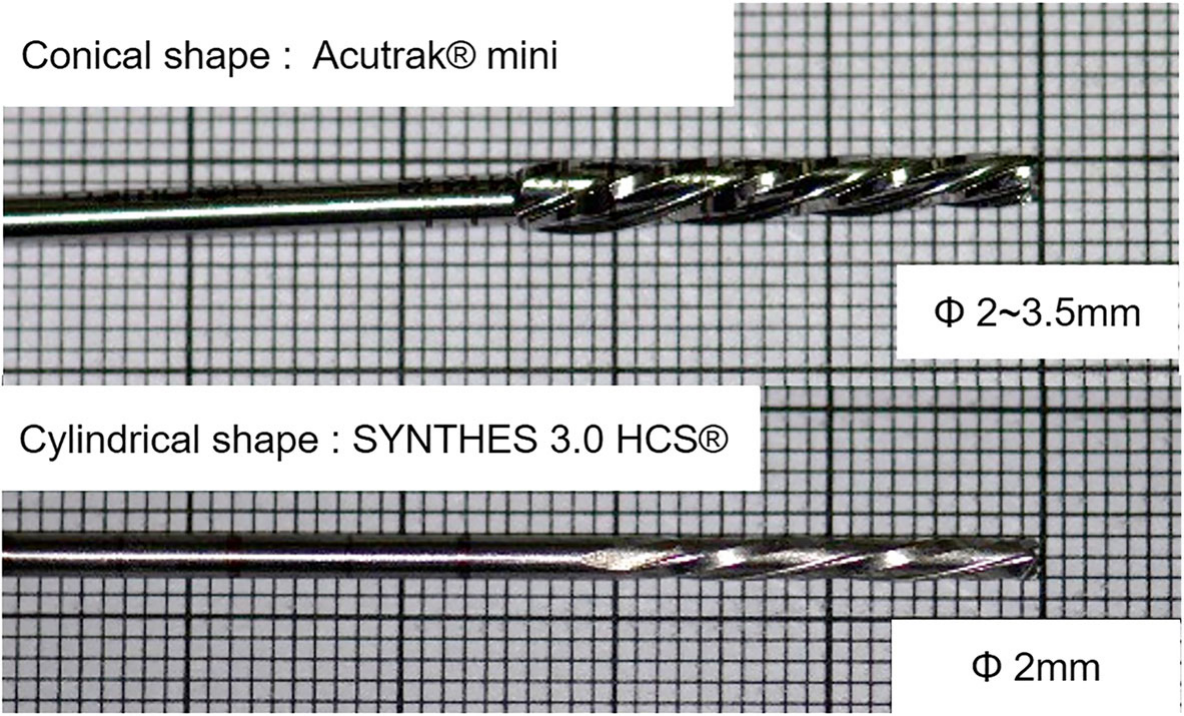
The Acutrak® mini drill bit has a conical type, where the diameter increases from 2.0 mm to 3.5 mm from the leading end to the trailing end. The Synthes 3.0 HCS® drill bit has a cylindrical type with a constant diameter of 2.0 mm

The Acutrak® mini is a titanium cannulated headless screw, which has a conical screw design with continuous threads over the entire length. The 24-mm screw has distal and proximal outer diameters of 2.8 mm and 3.5 mm, respectively, and the thread pitch gradually decreases from the distal end to the proximal end, where the screw tip has the widest thread pitch. A conical type drill bit is used for the Acutrak® mini; depth marks are on the surface of the drill bit. The screws tested in our study were the 24-mm long threaded Synthes 3.0 HCS® screw and the 24-mm Acutrak® mini (Fig. 1).

### Synthetic bone model

In previous studies that tested the compression forces generated by headless compression screws, cadaveric samples were used as a bone model. Limitations associated with the use of cadaveric bone include diverse intraosseous bone mineral density and potential individual differences between the cadavers [3]. Therefore, in this study, solid polyurethane foam (Sawbone®, grade 15 pcf, 0.24 g/cm3) with consistent density similar to cancellous bone was used for the scaphoid bone model [6].

### Experimental design

**Fig. 2:**
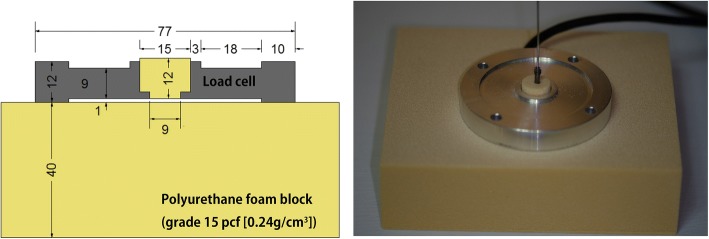
A load cell was placed between 12-mm- and 20-mm-thick Sawbone blocks (grade 15 pcf, [0.24 g/cm3]), which maintained a 1-mm space between the blocks. After placing the guide pin into the center of the block, drilling was performed to a predetermined depth, and the screws were inserted

A custom made load cell (DAISOCELL, Republic of Korea) was placed between the two Sawbone blocks (grade 15 pcf, 0.24 g/cm^3^), which were 12 mm and 40 mm in thickness, respectively; a 1-mm space between the blocks was maintained. Following placement of the guide pin into the center of the block and drilling to the predetermined depth, the 24-mm headless compression screw was inserted until its head entered the surface of the block. For the Synthes HCS®, the screw was inserted to the point when the researcher decided that the screw was fully compressed with a sleeve. Compression force was measured by the load cell immediately and 30 min after screw insertion (Fig. 2). Drilling depth of the Acutrak® mini ranged from 16 to 28 mm, in 2-mm increments. The drilling depth of the Synthes HCS® screw ranged from 22 to 28 mm in 2-mm increments. Compression force was measured twice at each depth, and then the mean value was calculated. The coefficient of regression and the coefficient of determination were calculated using SPSS version 23 (IBM, Armonk, NY, USA). The *p*-values < 0.05 were considered statistically significant.

## Results

**Fig. 3:**
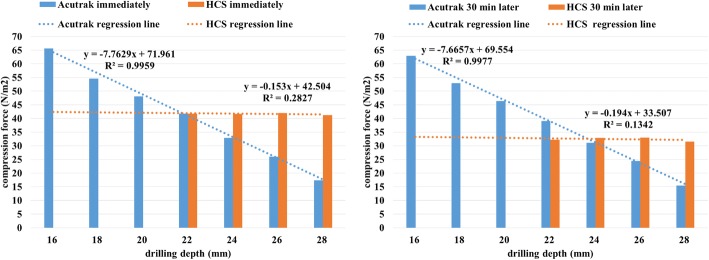
The Acutrak® mini showed a strong inverse correlation with the depth of drilling, while no significant inverse correlation was observed for the Synthes HCS® (R2, coefficient of determination)

The peak compression force of the Acutrak® mini was 65.66 N/m^2^ immediately after insertion and 62.92 N/m^2^ after 30 min at a drilling depth of 16 mm. The compression value gradually declined to 17.35 N/m^2^ and 15.48 N/m^2^, respectively, at a drilling depth of 28 mm. The compression force of the Acutrak® mini showed a strong inverse correlation with the depth of drilling (*r* = − 0.998, *p* < 0.001 immediately after insertion and *r* = − 0.999, *p* < 0.001 after 30 min; Fig. 3). Conversely, the peak compression force of the Synthes HCS® was 42.04 N/m^2^ immediately after insertion and 33.03 N/m^2^ after 30 min at a drilling depth of 26 mm, which subsequently decreased to 41.16 N/m^2^ and 31.56 N/m^2^ at a drilling depth of 28 mm. There was no significant inverse correlation between the compression force of Synthes HCS® and the drilling depth (*r* = − 0.532, *p* = 0.468 immediately after insertion and *r* = − 0.366, *p* = 0.634 after 30 min; Fig. 3). The Acutrak® mini provided greater compression force when drilled to a lesser depth, whereas the Synthes 3.0 HCS® showed minimal changes in the compression force regardless of the drilling depth.

## Discussion

Several studies have compared the compression force generated by headless compression screws; however, few studies investigated the exact method of screw insertion [4, 7, 9]. Based on our findings, the drilling depth is important for the full-threaded Acutrak® screw with a conical type drill bit, and full description of the insertion method is necessary because the drilling depth can be a critical factor for determining the results of the study.

Our study showed that at a drilling depth of 16 to 28 mm, the 24-mm Acutrak® mini demonstrated a 3.88 N/m^2^ decrease in compression force for every 1 mm increase in drilling depth, which was supported by the strong negative correlation between the drilling depth and compression force (*r* = − 0.998, *p* < 0.001). By comparison, the compression force generated by the 24-mm Synthes 3.0 HCS® screw decreased by 0.077 N/m^2^ for every 1-mm increase in drilling depth (*r* = − 0.532, *p* = 0.468). Although the Acutrak® mini generated greater compression force compared to the Synthes 3.0 HCS®, the compression force generated by the Acutrak® mini decreased significantly as the drilling depth increased. The immediate compression force generated by the Acutrak® mini ultimately declined to the same compression force generated by the Synthes 3.0 HCS® when drilled to a depth of 21.9 mm, but reached the same compression force after 30 min as the Synthes 3.0 HCS® when drilled to a depth of 23.6 mm. Therefore, our results indicated that the Acutrak® mini generated greater compression force compared to the Synthes 3.0 HCS® when drilled to a depth of less than 24 mm.

In addition, differences in compression force measured immediately after screw insertion and at 30 min post-insertion was less for the Acutrak® mini than the Synthes 3.0 HCS®. Because the Acutrak® mini has continuous threads throughout the entire length of the screw, a greater extent of total thread area ultimately generates a greater bearing capacity in cancellous bone. The result also suggested that while the compression force generated by the Acutrak® mini was generated by the design of the screw itself, the compression force generated by the sleeve was not completely maintained by the screw in the case of the Synthes 3.0 HCS®.

Taken together, our results indicated that a cylindrical type drill bit could maintain a constant degree of compression regardless of the drilling depth, while a conical type drill bit could control compression force according at any drilling depth. While this characteristic of the conical type drill bit is an advantage, it also is a disadvantage because of the significant decline in compression force when over-drilling.

**Fig. 4:**
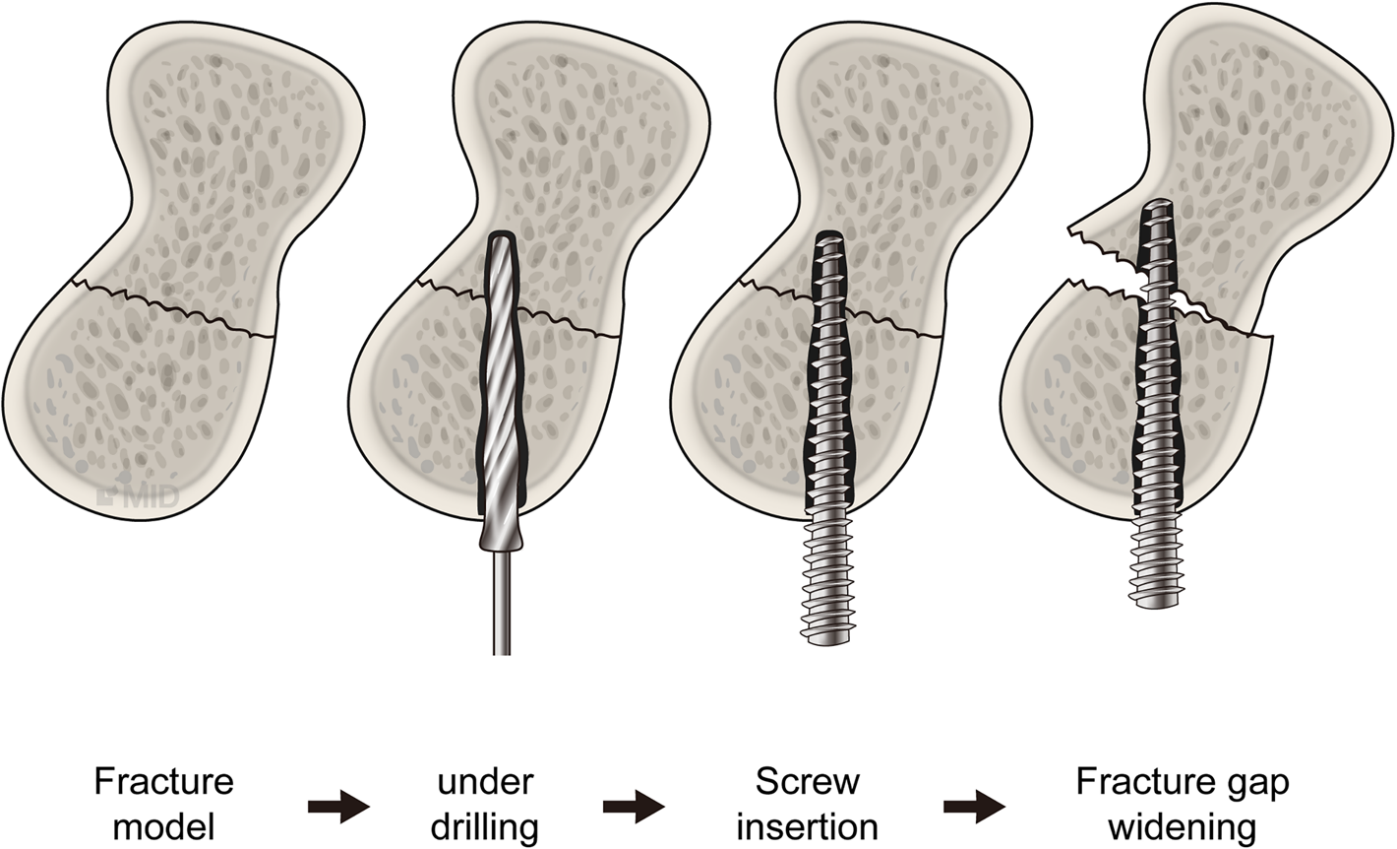
When under-drilling, if subcortical bone is present in the leading direction of a screw, the advancing screw can push the fracture area, widening the fracture gap

Therefore, when a strong compression force is needed, a screw with a conical type drill bit can be used with under-drilling. However, when subcortical bone is present in the leading direction of a screw, the screw can push the fracture area as it advances and result in the fracture space widening, in spite of the drilling depth (Fig. 4). Furthermore, if the size of the target bone fragment is small, the bone may crack, being unable to tolerate the increasing diameter of the inserting screw. A small-sized bone, such as the scaphoid, may also have unpredictable subcortical bone in the leading direction of a screw, depending on the screw insertion angle. Therefore, when using a conical type drill bit, an accurate and careful insertion of the guide wire is needed, and the precise drilling depth must be determined. When a cylindrical type drill bit is used, the compression force generated may be less, but the drilling action is relatively simpler. Conversely, when an intra-articular fracture of large sized joints, such as the knee joint or the shoulder joint, needs reduction, a conical type drill bit may have the advantage of generating greater compression force with under-drilling, because the distal part of the screw meets a wide cancellous bone.

The limitation of our study was that conical and cylindrical type drill bits were used specifically for the Acutrak® mini and the Synthes 3.0 HCS®, respectively, and the effect of the drill bit in the generated compression force could not be evaluated independently from the screw design. Secondly, a polyurethane sawbone model cannot perfectly simulate the actual bone structure, which is an important consideration when cancellous bone is surrounded by cortical bone and subcortical bone is present in an actual fracture. In addition, actual bone has highly variable bone mineral density according to its location; therefore, our results may not be easily translated to clinical practice, when applied to actual bone.

## Conclusion

If the screw length and the drill depth are the same, the Synthes 3.0 HCS® (cylindrical type drill bit) is safer and easier to use as it has no change in the compression force even when over-drilling because the compression force of the two screws is similar. As for the Acutrak® mini (conical type drill bit), while it is technically demanding due to varying compression force according to the drill depth, it can be used in certain cases because it can give stronger compression force through under-drilling.

## Abbreviations

r: Correlation coefficient
R^2^: Coefficient of determination
